# Activatable Fluorescence Imaging of Macrophages in Cerebral Aneurysms Using Iron Oxide Nanoparticles Conjugated With Indocyanine Green

**DOI:** 10.3389/fnins.2020.00370

**Published:** 2020-04-22

**Authors:** Hiroyuki Ikeda, Akira Ishii, Kohei Sano, Hideo Chihara, Daisuke Arai, Yu Abekura, Hidehisa Nishi, Masahiro Ono, Hideo Saji, Susumu Miyamoto

**Affiliations:** ^1^Department of Neurosurgery, Kyoto University Graduate School of Medicine, Kyoto, Japan; ^2^Department of Patho-Functional Bioanalysis, Graduate School of Pharmaceutical Sciences, Kyoto University, Kyoto, Japan; ^3^Laboratory of Biophysical Chemistry, Kobe Pharmaceutical University, Kobe, Japan

**Keywords:** macrophage, inflammation, cerebral aneurysm, iron oxide nanoparticles, fluorescent imaging, indocyanine green

## Abstract

**Background and Purpose:**

Chronic inflammation is involved in the formation and enlargement of cerebral aneurysms (CAs), with macrophages playing a key role in the process. The present study evaluated visualization of macrophages present in CAs using an activatable fluorescent probe (IONP-ICG) comprising an iron oxide nanoparticles (IONPs) conjugated with indocyanine green (ICG).

**Methods:**

IONP-ICG was intravenously administered to 15-week-old CA model rats (*n* = 8), and *ex vivo* near-infrared fluorescence (NIRF) imaging and histological assessment of exposed CAs and cerebral arteries were performed 48 h later. Similar evaluations were performed in the control group, which included CA model rats given IONPs or ICG (*n* = 8 each).

**Results:**

ICG-derived NIRF signals were detected in three IONP-ICG group rats but not in IONP or ICG control groups. Among the three rats that exhibited signals, NIRF signal accumulation was observed in the CA of two rats and at the site of hemodynamic stress in the left posterior cerebral artery in one rat. Histologically, NIRF signals correlated strongly with macrophage localization. A total of 13 CAs formed in the IONP-ICG group. The number of macrophages in the CA wall was significantly greater in the two CAs that exhibited NIRF signals compared to the remaining 11 CAs that did not (*P* = 0.037). Moreover, all 11 CAs that did not exhibit NIRF signals were iron-negative, while the two CAs that exhibited NIRF signals were both iron-positive (*P* = 0.013).

**Conclusion:**

NIRF imaging using an activatable IONP-ICG probe is feasible for detecting the macrophage-rich regions in CAs and the cerebral artery wall, which is considered an early lesion in the process of CA formation.

## Introduction

Chronic inflammation is involved in the formation and enlargement of cerebral aneurysms (CAs), and macrophages, which are the primary inflammatory cells that accumulate in the aneurysm wall, play an essential role in these processes (Aoki et al., 2007a, b, 2017a; Kanematsu et al., 2011; Kataoka, 2015). Moreover, a greater number of macrophages are known to be present in the aneurysm wall of ruptured CAs compared to unruptured, suggesting an association between macrophages and CA rupture (Frosen et al., 2004). For this reason, macrophage imaging may enable identification of early lesions in the CA formation process as well as prediction of enlargement and rupture. As a molecular imaging method that detects chronic inflammation, near-infrared fluorescence (NIRF) has been gaining attention. Specifically, NIRF probes are highly sensitive, offer relatively good tissue permeability, present minimal effects from autofluorescence, and exhibit quantifiable fluorescence intensity (Deguchi et al., 2006; Kaijzel et al., 2010). However, effective preclinical NIRF probes have yet to be reported in the CA field.

Iron oxide nanoparticles (IONPs) have been widely utilized *in vivo* due to their high biocompatibility and low toxicity (Arami et al., 2015), and the ultrasmall superparamagnetic IONP ferumoxytol (AMAG Pharmaceuticals, Lexington, MA, United States) gained approval from the United States Food and Drug Administration in 2009 (Lu et al., 2010). Since intravenously administered ferumoxytol is non-specifically taken up by macrophages, IONPs accumulate at inflammatory sites where macrophages are present, thereby changing the local magnetic field (Hasan et al., 2012; Aoki et al., 2017b). This property suggests its potential for use in magnetic resonance imaging, enabling non-invasive imaging of macrophages accumulating in the CA of humans. On the other hand, indocyanine green (ICG), another biocompatible and minimally toxic substance (Cherrick et al., 1960), is the only NIRF dye approved by the United States Food and Drug Administration to be used for clinical purposes. For these reasons, a probe incorporating IONPs and ICG may offer a highly biocompatible NIRF probe that targets macrophages accumulating in CAs.

When multiple fluorescent molecules are in proximity to each other, their interaction leads to fluorescence self-quenching (Hama et al., 2007). Moreover, interactions between ICG and carrier molecules can lead to auto-quenching (Ogawa et al., 2009). Considering these properties, it is possible that ICG-conjugated carrier molecules could be used as target cell-specific activatable probes with high background signal ratios. Since CAs are relatively small targets, detection of chronic inflammation using a target cell-activatable probe would be diagnostically useful. We previously synthesized a NIRF probe (IONP-ICG) comprising IONPs conjugated with ICG and demonstrated its potential as an activatable probe which targets macrophages (Ikeda et al., 2018). In particular, we confirmed macrophage uptake of quenched IONP-ICG *in vitro* and intracellular activation of NIRF. Furthermore, IONP-ICG synthesized by mixing with a molecular ratio of 1:20 IONP:ICG enabled NIRF imaging of macrophages in atherosclerotic plaques with the greatest background ratio. Therefore, we postulated that NIRF imaging using an IONP-ICG probe might enable targeted visualization of CA macrophages, facilitating prediction of the formation, enlargement, and rupture of CAs. The present study evaluated whether macrophages localized to CAs can be specifically detected using our activatable IONP-ICG NIRF probe.

## Materials and Methods

### Materials

IONPs coated with dextran (nanomag^®^-D-spio; diameter, 20 nm; mean molecular weight, 3500 kDa; amino groups on particle surface) were purchased from Corefront Co. (Tokyo, Japan). ICG-EG4-Sulfo-OSu was purchased from Dojindo Molecular Technologies (Kumamoto, Japan). Methoxypolyethylene glycol (PEG) succinate *N*-hydroxysuccinimide (NHS) (PEG-NHS-ester; molecular weight, 2 kDa; SUNBRIGHT^®^ ME-020CS) was purchased from NOF America Co. (White Plains, NY, United States). ICG was purchased from Tokyo Chemical Industry Co., Ltd. (Tokyo, Japan).

### Synthesis of IONP-ICG and IONPs Conjugated With PEG (IONP-PEG)

Synthesis of IONP-ICG was performed according to our previous report (Ikeda et al., 2018). Briefly, IONP-ICG was synthesized by mixing the IONP nanomag^®^-D-spio (2.4 mg Fe, 1.43 nmol) and ICG-EG4-Sulfo-OSu (8.5 mmol/L in dimethyl sulfoxide) in a 1:20 IONP:ICG molecular ratio (ICG-EG4-Sulfo-OSu, 28.6 nmol). The concentration of IONPs and ICG in synthesized IONP-ICG was determined by measuring the absorption at 535 and 768 nm using a Shimadzu UV-Vis NIR system (UV-1800; Kyoto, Japan) to determine the number of ICG molecules conjugated to each IONP. As a control probe, IONP-PEG was synthesized by mixing the IONP nanomag^®^-D-spio (2.4 mg Fe, 1.43 nmol) and PEG-NHS-ester (1.43 mg, 715 nmol) to quench the amino groups of the particles.

### Animal Models

All animal experiments were conducted following institutional guidelines and were approved by the Kyoto University Animal Care Committee. CA model rats were prepared as described previously (Hashimoto et al., 1980), with some modifications. CAs were formed by inducing hemodynamic stress through ligation of the left renal artery and left common carotid artery in 7-week-old male Sprague Dawley rats (Japan SLC, Shizuoka, Japan). These surgeries were conducted under general anesthesia with intraperitoneal injection of 50 mg/kg pentobarbital. Rats were fed chow containing 8% NaCl and 0.12% 3-aminopropionitrile (Tokyo Chemical), an inhibitor of lysyl oxidase that catalyzes the cross-linking of collagen and elastin, for 8 weeks. Furthermore, 3-aminopropionitrile fumarate (1000 mg/kg; Tokyo Chemical) was injected intraperitoneally four times at weekly intervals immediately after surgery to promote enlargement of CAs. At 15 weeks, IONP-ICG group rats (*n* = 8) were intravenously injected with IONP-ICG (27.9 mg Fe/kg, 0.5 mmol Fe/kg, 16.6 nmol IONP-ICG/kg) via the tail vein. The mean number of ICG molecules conjugated to each IONP in IONP-ICG was 7.5 ± 0.6 in the present study. Control group rats were injected with either IONP-PEG (27.9 mg Fe/kg, 0.5 mmol Fe/kg, 16.6 nmol IONP-PEG/kg; *n* = 8) or ICG (96.6 μg ICG/kg, 125 nmol ICG/kg; *n* = 8). In total, 24 rats were used in the present study (*n* = 8 per group).

### Biodistribution Study

48 h after probe administration, rats were deeply anesthetized with an intraperitoneal injection of 50 mg/kg pentobarbital and perfused transcardially with 4% paraformaldehyde after 500 μL of blood was collected from the heart to measure the fluorescence intensity. After euthanasia by cervical dislocation and decapitation, the brain along with cerebral arteries, liver, spleen, kidneys, lungs, heart, and muscles were dissected. Cerebral arteries were stripped from the brain after the microscopic observation described below. Fluorescence images of tissues were acquired using an IVIS Imaging System 200 (excitation/emission, 745/820 nm; exposure time, 1 s; PerkinElmer, Waltham, MA, United States) to determine the distribution of ICG. Similar-sized regions of interest were circled within respective tissues, and the mean fluorescence intensity (p/s/cm^2^/sr) of each region of interest after subtraction of background fluorescence was measured.

### NIRF Imaging

Excised brains were washed with 0.04% bromophenol blue solution to facilitate visualization of cerebral arteries by visible light imaging then immediately washed with phosphate-buffered saline. After, brains were imaged *ex vivo* using a Nuance EX multispectral imaging camera (PerkinElmer) mounted on an MVX10 macro zoom fluorescence stereomicroscope (Olympus, Tokyo, Japan) equipped with a Cy7 filter set (excitation/emission, 670–745/776 nm long-pass). NIRF images of the brain were acquired by multispectral imaging at 10-nm wavelength increments from 770 to 950 nm with an exposure time of 5 s. Nuance version 3.0.2 software (PerkinElmer) was used to examine the fluorescence signals derived from ICG by measuring the fluorescence spectra, unmixing the autofluorescence spectra of the tissues, and eliminating background noise. Visible light and NIRF imaging were performed from the ventral side of the brain. Cerebral arteries as well as whole circle of Willis arterial rings stripped from the brain underwent visible light and NIRF imaging.

### Histology and Fluorescence Microscopy

Cerebral aneurysm was defined as bulging or saccular shape of the cerebral artery wall with disruption of the internal elastic lamina. CAs were searched by stereomicroscopy from the ventral side of the brain and from both ventral and dorsal sides of cerebral arteries stripped from the brain. Bifurcations of the right anterior cerebral and olfactory arteries, including the CAs, as well as stereomicroscopy-detected CAs in the area around the circle of Willis arterial rings were carefully excised. Two or more CAs arising side-by-side at the same location were evaluated separately. In addition, if CAs could not be detected by stereomicroscopy, arteries showing NIRF signals derived from ICG were still excised. Cryosections (thickness, 10 μm) were obtained from freshly frozen samples. ICG NIRF signals were observed using a BZ-X710 fluorescence microscope (KEYENCE, Osaka, Japan) equipped with a filter for ICG (excitation/emission, 775–825/845–900 nm). Immunohistological staining was then performed using an anti-mouse ionized calcium binding adapter molecule 1 (Iba1) antibody (Wako Pure Chemical Industries, Osaka, Japan) for localization of macrophages. Iron staining was performed using a Berlin blue staining set (Wako Pure Chemical Industries). Elastica van Gieson (EVG) staining was performed to assess disruption of the internal elastic lamina. Light microscopy was performed using an FSX100 microscope (Olympus).

Morphological evaluation of CAs was performed as described previously (Ikedo et al., 2017), with some modifications. CA size was defined as the mean maximum height and width including the aneurysm wall. Aneurysm wall thickness was measured at the tip of the CA. Cell counting was performed as described previously (Aoki et al., 2009), with some modifications. Macrophages within the aneurysm wall were manually counted under 40 × magnification in a selected circular field (diameter, 100 μm) at the tip of the CA. The same lower threshold value was set for obtained images using cellSens Dimension software (Olympus) to determine whether cells were immunostain-positive or not; detected cells were defined as positive. For iron staining, blue spots within the CA wall corresponding to iron deposition were defined as iron-positive.

### Statistical Analysis

Statistical analyses were performed using JMP version 11 software (SAS Institute, Cary, NC, United States). Quantitative data were expressed as the mean ± standard deviation. Fisher’s exact test between two groups and Pearson’s Chi-square test between three groups were used to examine categorical variables. The Mann-Whitney *U*-test between two groups and Kruskal-Wallis test followed by the Steel-Dwass test between three groups or more were used for continuous variables. A *P* < 0.05 was considered statistically significant.

## Results

### Biodistribution Study

**FIGURE 1:**
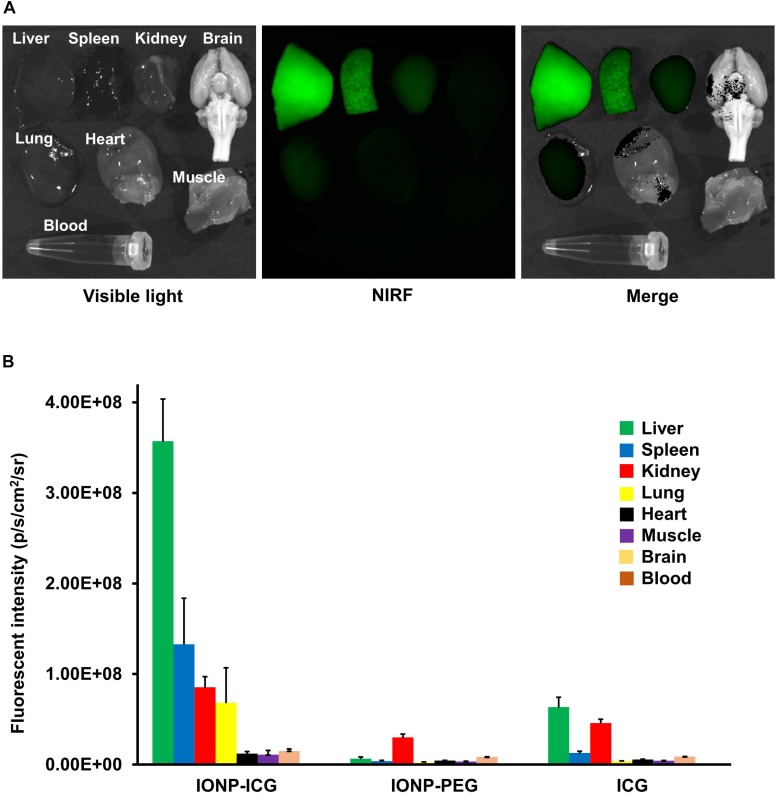
Representative images after IONP-ICG administration and biodistribution of NIRF signals 48 h after administration of probes. **(A)** Representative images of visible light, NIRF, and merged imaging of the liver, spleen, kidney, lung, heart, muscle, brain, and blood after IONP-ICG administration. **(B)** Graphical representation of NIRF signal intensity in each organ after administration of IONP-ICG, IONP-PEG, and ICG (*n* = 8 each). All values are expressed as the mean ± standard deviation.

The results of biodistribution studies 48 h after probe administration are summarized in Figure 1. In the IONP-ICG group, the greatest accumulation of NIRF signal was in the liver, followed by the spleen, kidney, and lung. NIRF signal intensity was significantly lower in the heart, muscle, brain, and blood (*P* < 0.05). In the IONP-PEG group, the NIRF signal was highest in the kidney, with barely any detected in other organs. In the ICG group, the NIRF signal was most intense in the liver followed by the kidney; accumulation was negligible in other organs. Comparison of each organ between the three groups revealed significantly greater NIRF signal accumulation in the IONP-ICG compared to IONP-PEG and ICG groups for all organs except the blood (*P* < 0.001). NIRF signal intensities in the blood were extremely low in all three groups and did not differ significantly between them (*P* = 0.11).

### NIRF Imaging

**FIGURE 2:**
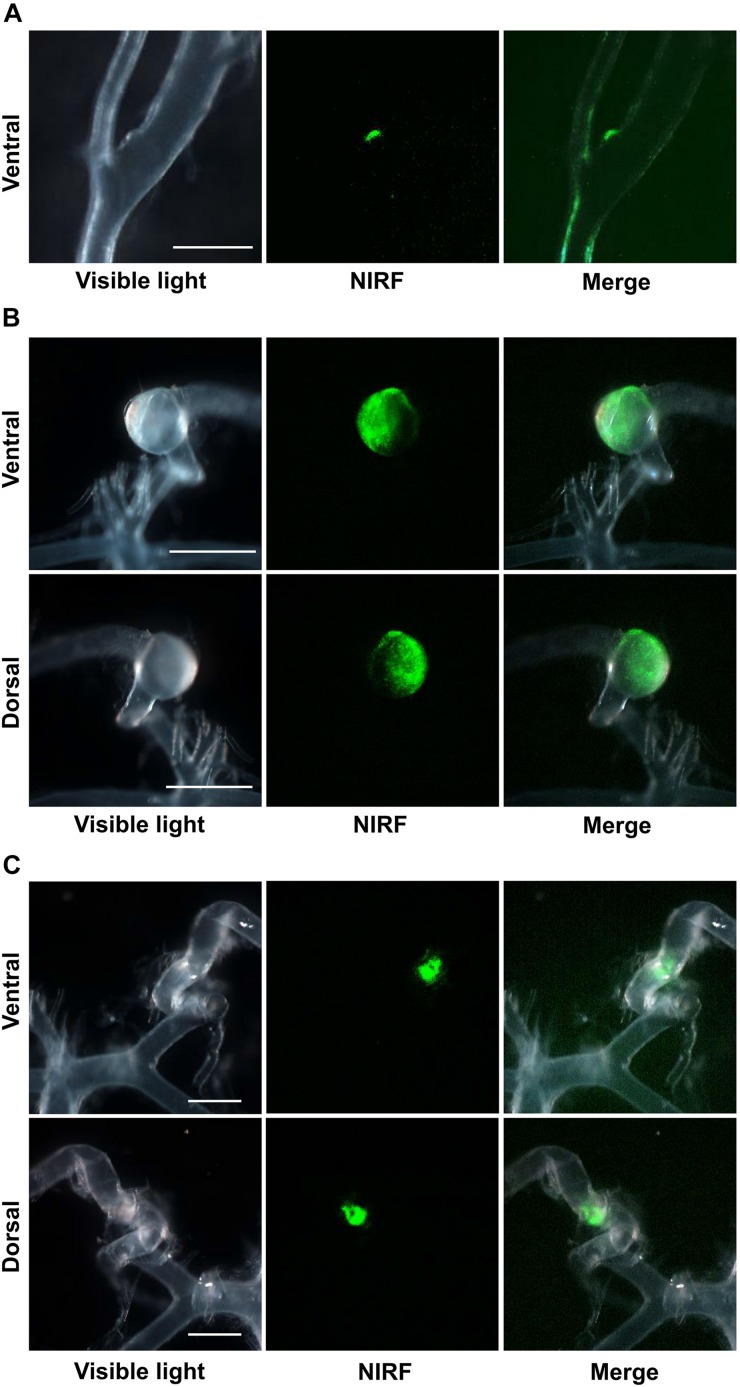
NIRF imaging of excised CAs and cerebral arteries. **(A)** Visible light, NIRF, and merged NIRF and visible light images from the ventral side of NIRF-case 1. NIRF signals were observed at the bulging CA that formed along the right anterior cerebral artery at a site distal to the bifurcation of the right anterior cerebral and olfactory arteries. Scale bar, 500 μm; original magnification, 25×. **(B)** Visible light, NIRF, and merged NIRF and visible light images from both ventral and dorsal sides of NIRF-case 2. NIRF signals were observed in the saccular CA that formed at the bent portion of the left posterior cerebral artery. An abundance of spotted NIRF signals was observed at the aneurysm wall on the larger curvature side of the CA, while NIRF signal was hardly detected at the CA wall on the smaller curvature side. Scale bars, 1 mm; original magnification, 25×. **(C)** Visible light, NIRF, and merged NIRF and visible light images from both ventral and dorsal sides of NIRF-case 3. NIRF signals were localized to the bent portion of the left posterior cerebral artery, and a CA had not formed at this site. Scale bars, 500 μm; original magnification, 25×.

**FIGURE 3:**
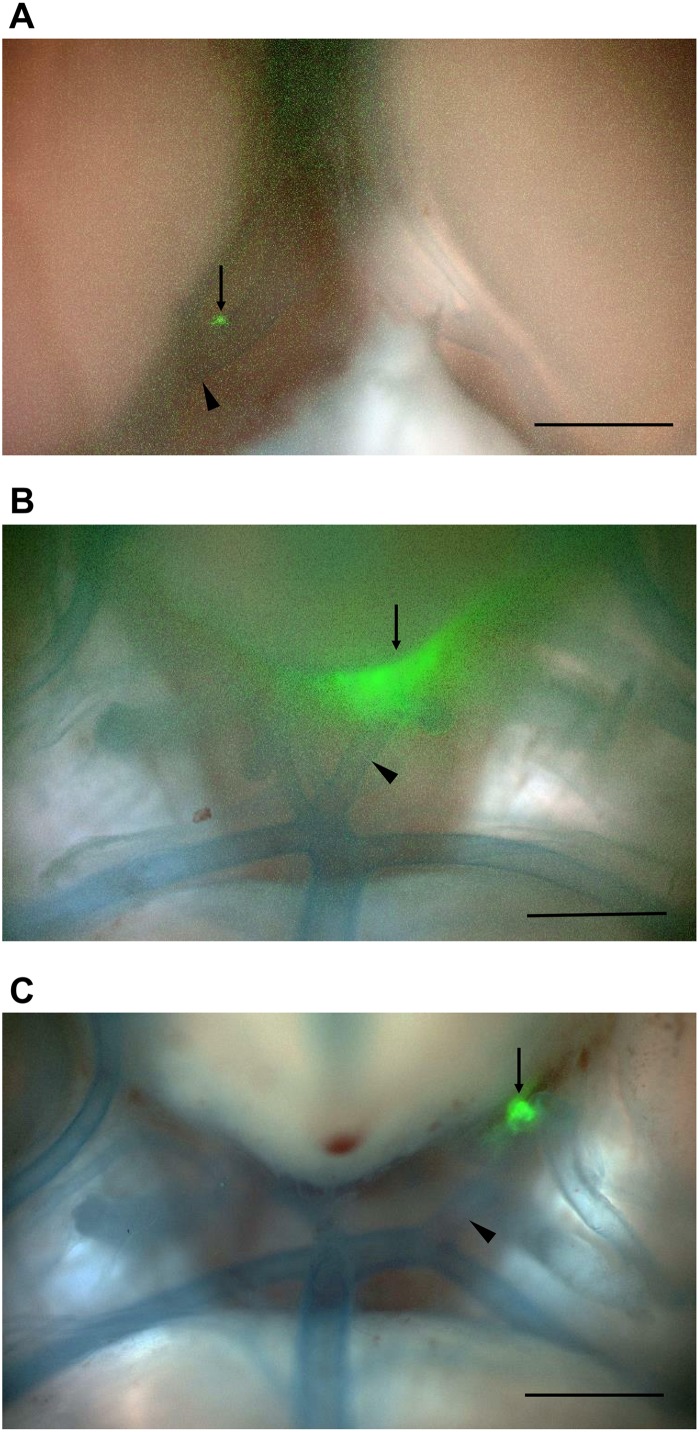
NIRF imaging of CAs and cerebral arteries with the brain in the background. Merged NIRF and visible light images from the ventral side. **(A)** NIRF signals in the bulging CA (arrow) that formed along the right anterior cerebral artery at a site distal to the bifurcation (arrowhead) of the right anterior cerebral and olfactory arteries in NIRF-case 1 were detected. **(B)** NIRF signals in the saccular CA (arrow) that formed at the bent portion of the left posterior cerebral artery (arrowhead) in NIRF-case 2 were detected. **(C)** Localized NIRF signals at the bent portion (arrow) of the left posterior cerebral artery (arrowhead) in NIRF-case 3 were clearly detected. Scale bars, 1 mm; original magnification, 25×.

ICG-derived NIRF signals were detected in the CA or cerebral artery of three IONP-ICG rats by NIRF imaging. In contrast, ICG-derived NIRF signals were not detected in IONP-PEG or ICG groups. Figures 2, 3 show stereomicroscopic findings from the three rats that exhibited NIRF signals (“NIRF-cases 1–3”). In NIRF-case 1, NIRF signal was observed at a bulging CA that formed along the right anterior cerebral artery distal to the bifurcation of the right anterior cerebral and olfactory arteries (Figure 2A). NIRF signal could be detected when this CA was examined with the brain in the background (Figure 3A). In NIRF-case 2, NIRF signal was observed at a saccular CA formed at the bent portion of the left posterior cerebral artery. NIRF signals accumulated abundantly in a spotted manner at the aneurysm wall of the larger curvature of the CA, with negligible detection on the aneurysm wall of the smaller curvature of the CA (Figure 2B). NIRF signal could be detected when this CA was examined with the brain in the background (Figure 3B). In NIRF-case 3, NIRF signal was localized at a bent portion of the left posterior cerebral artery, and no CA had formed at this site according to stereomicroscopic examination (Figure 2C). When this cerebral artery was examined with the brain in the background, the NIRF signal in the cerebral artery was clearly detectable (Figure 3C).

### Morphological and Histological Findings of CAs

Microscopic and histological findings detected CAs in all 24 rats included in the study, amounting to 38 total CAs. Of them, 13 were in the IONP-ICG group, 13 in the IONP-PEG, and 12 in the ICG group. Table 1 shows the details of morphological and histological findings of all CAs within each group. Of the 38 CAs, 33 formed at the bifurcation of the right anterior cerebral and olfactory arteries, four at the left posterior cerebral artery, and one at the anterior communicating artery. All saccular CAs formed at the left posterior cerebral artery, with one in the IONP-ICG group, and three in the IONP-PEG; saccular CAs did not form in the ICG group.

**TABLE 1:** Morphological and histological evaluations of cerebral aneurysms.

	Total (*n* = 24)	IONP-ICG (*n* = 8)	IONP-PEG (*n* = 8)	ICG (*n* = 8)
Aneurysm	38	13	13	12
Saccular aneurysm	4	1	3	0
Mean aneurysm size (μm)	150 ± 190	130 ± 176	208 ± 257	101 ± 60
Mean aneurysm wall thickness (μm)	17.2 ± 22.7 (*n* = 37)	16.8 ± 5.6	22.5 ± 33.8 (*n* = 12)	11.5 ± 3.5
Mean number of macrophages	6.6 ± 7.5	5.8 ± 6.3	10.0 ± 2.8	3.8 ± 2.0
Iron positivity	5 (13%)	2 (15%)	3 (23%)	0 (0%)

*In IONP-ICG, IONP-PEG, and ICG groups, the numbers of cerebral aneurysms found were 13, 13, and 12, respectively. Saccular aneurysms were observed in IONP-ICG and IONP-PEG groups. The aneurysm size, aneurysm wall thickness, and number of macrophages in cerebral aneurysms were measured. These values are shown as the mean ± standard deviation. Numbers in parentheses (iron positivity) correspond to percentages of the total number of cerebral aneurysms in that group. None of the items listed in this Table differed significantly between individual groups. IONP-ICG, iron oxide nanoparticles (IONPs) conjugated with indocyanine green (ICG); IONP-PEG, IONPs conjugated with methoxypolyethylene glycol (PEG).*

Mean CA size was 150 ± 190 μm (range, 41–1028 μm) overall, with 130 ± 176 μm (range, 41–710 μm) in the IONP-ICG group, 208 ± 257 μm (range, 48–1028 μm) in the IONP-PEG group, and 101 ± 60 μm (range, 43–247 μm) in the ICG group. The mean size of the four saccular CAs at the left posterior cerebral artery was 573 ± 366 μm (range, 268–1028 μm), which was significantly greater than that of bulging CAs (97 ± 48 μm; range, 41–247 μm; *P* = 0.0013). Mean CA wall thickness was 17.2 ± 22.7 μm (range, 6–118 μm) overall, with 16.8 ± 5.6 μm (range, 7–83 μm) in the IONP-ICG group, 22.5 ± 33.8 μm (range, 6–118 μm) in the IONP-PEG group, and 11.5 ± 3.5 μm (range, 6–18 μm) in the ICG group. One saccular CA in the left posterior cerebral artery in the IONP-PEG group did not have a lumen, preventing measurement of CA wall thickness. Therefore, this CA was excluded from the calculation of overall mean CA wall thickness. The mean wall thickness of three saccular CAs of the left posterior cerebral artery was 88.7 ± 30.0 μm (range, 65–118 μm), which is significantly thicker than that of bulging CAs (10.9 ± 3.4 μm; range, 6.0–18.0 μm; *P* = 0.0047).

The mean number of macrophages was 6.6 ± 7.5 (range, 0–36) overall, with 5.8 ± 6.3 (range, 0–20) in the IONP-ICG group, 10.0 ± 2.8 (range, 1–36) in the IONP-PEG group, and 3.8 ± 2.0 (range, 1–7) in the ICG group. The mean number of macrophages in the four saccular CAs of the left posterior cerebral artery was 24.3 ± 8.0 (range, 18–36), which was significantly greater than that of bulging CAs (4.5 ± 3.6; range, 0–20; *P* = 0.0014). An iron-positive wall was observed in five CAs (13%) overall, with two (15%) in the IONP-ICG group, three (23%) in the IONP-PEG, and 0 (0%) in the ICG group. All four saccular CAs of the left posterior cerebral artery were iron-positive. None of the items listed in Table 1 differed significantly between individual groups (number of CAs, *P* = 0.99; number of saccular CAs, *P* = 0.16; CA size, *P* = 0.15; CA wall thickness, *P* = 0.98; number of macrophages, *P* = 0.13; iron positivity, *P* = 0.22).

### Histological Findings of Lesions With NIRF Signals

**FIGURE 4:**
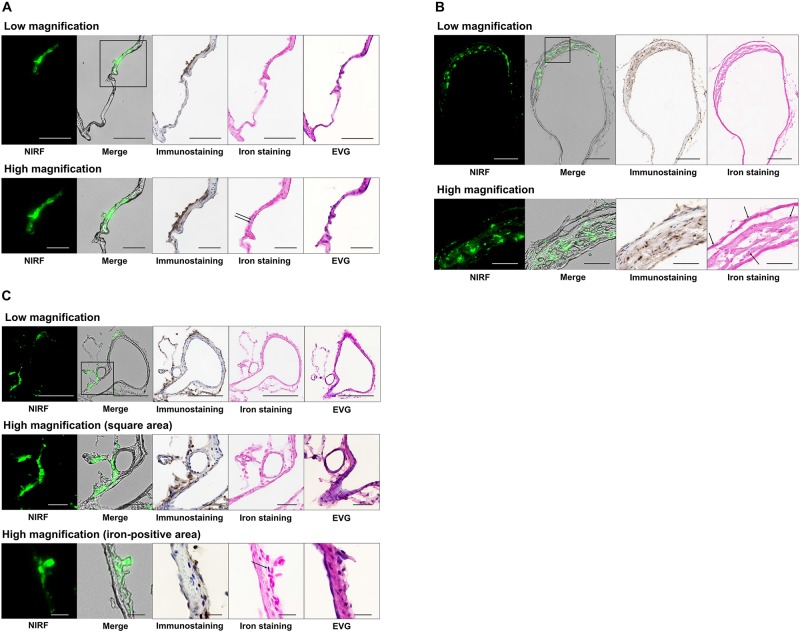
Histology and fluorescence microscopy of CAs and cerebral arteries with NIRF signals. NIRF, merged NIRF and bright-field images, Iba1 immunostaining, iron staining, and EVG staining were performed. High magnification images other than the iron-positive area of **(C)** depict the square region within the low magnification images. **(A)** CA in NIRF-case 1. NIRF signals correlated very well with the presence of macrophage infiltration (Iba1 immunostaining) in the CA. Iron staining showed blue areas (arrows) at the CA wall with a high NIRF signal. EVG staining showed continuous disruption of the internal elastic lamina from the bifurcation of the right anterior cerebral and olfactory arteries to the distal side of the distal CA wall. Scale bars: low magnification, 100 μm; high magnification, 50 μm. **(B)** CA in NIRF-case 2. NIRF signals correlated very well with the presence of macrophage infiltration in the CA. Iron staining showed very small blue areas (arrows) scattered within the thickened CA wall. Scale bars: low magnification, 200 μm; high magnification, 50 μm. **(C)** Left posterior cerebral artery in NIRF-case 3. NIRF signals correlated very well with the presence of macrophage infiltration in the adventitia of the cerebral artery. Iron staining shows a small number of very small blue areas (arrow) at the adventitia. EVG staining did not detect any disruption of the internal elastic lamina of the artery wall. Scale bars: low magnification, 200 μm; high magnification (square area), 50 μm; high magnification (iron-positive area), 20 μm.

Figure 4 shows histological findings for the three rats with NIRF signals in a CA or cerebral artery. In NIRF-case 1, a very small CA formed at the bifurcation of the right anterior cerebral and olfactory arteries, and a bulging CA formed along the right anterior cerebral artery distal to the very small CA. Fluorescence microscopic examination of an unstained section detected NIRF signal at the wall of the distal CA with the strongest intensity at its tip. This NIRF signal in the CA wall correlated strongly with the localization of Iba1-positive macrophages. Iron staining showed blue areas in the CA wall with high NIRF signal. EVG staining revealed continuous disruption of the internal elastic lamina from the bifurcation of the right anterior cerebral and olfactory arteries to the distal side of the distal CA (Figure 4A). In NIRF-case 2, the wall on the larger curvature side of the saccular CA was thicker than the artery wall, but the CA wall on the smaller curvature side had not thickened. Fluorescence microscopy of the unstained section showed an abundance of spotted NIRF signals at the thickened CA wall, but negligible signals at the CA wall that had not thickened. The NIRF signal in the CA wall correlated strongly with Iba1-positive macrophage localization, and iron staining showed very small blue areas scattered within the thickened CA wall (Figure 4B). In NIRF-case 3, fluorescence microscopy of the unstained section showed NIRF signals localized to the adventitia of the artery wall. Iba1-positive macrophages accumulated along the entire circumference of the artery wall adventitia. The NIRF signal of the artery wall correlated strongly with Iba1-positive macrophage localization, and iron staining showed a few very small blue areas distributed at the artery wall adventitia. EVG staining did not detect disruptions of the internal elastic lamina of the artery wall (Figure 4C).

To determine the type of CAs that exhibited NIRF signals, the two CAs in the IONP-ICG group with NIRF signals were compared with the 11 CAs of this group without signals (Table 2). Although the mean size of CAs with NIRF signals (399 μm; actual size, 89, 710 μm) was obviously larger than those without (81 ± 29 μm; range, 41–120 μm), the difference was no significant (*P* = 0.28). The mean wall thickness of CAs with and without NIRF signals was 49.0 μm (actual thickness, 15, 83 μm) and 10.9 ± 3.9 μm (range, 7–18 μm), respectively; this difference was not significant (*P* = 0.092). On the other hand, the mean number of macrophages in CAs with NIRF signals (19.0; actual numbers, 18, 20) was significantly different from those without signals (3.4 ± 2.3; range, 0–7; *P* = 0.037). Furthermore, both CAs with NIRF signals showed iron-positive staining, while all 11 CAs without signals were iron-negative, indicating that CAs with NIRF signals were significantly more likely to be iron-positive (*P* = 0.013).

**TABLE 2:** Relationship between cerebral aneurysms with and without near-infrared fluorescence (NIRF) signals after administration of IONP-ICG.

	Total aneurysm (*n* = 13)	NIRF (+) (*n* = 2)	NIRF (−) (*n* = 11)	*P*-value
Saccular aneurysm	1 (8%)	1 (50%)	0 (0%)	0.15
Mean aneurysm size (μm)	130 ± 176	399 [89, 710]	81 ± 29	0.28
Mean aneurysm wall thickness (μm)	16.8 ± 5.6	49.0 [15, 83]	10.9 ± 3.9	0.092
Mean number of macrophages	5.8 ± 6.3	19.0 [18, 20]	3.4 ± 2.3	0.037
Iron positivity	2 (15%)	2 (100%)	0 (0%)	0.013

*Among cerebral aneurysms (n = 13) found in IONP-ICG group, there were two cerebral aneurysms with NIRF signals. The aneurysm size, aneurysm wall thickness, and number of macrophages in cerebral aneurysms were measured. These values are shown as the mean ± standard deviation except for NIRF (+) group. In NIRF (+) group, actual values measured are described in brackets. Numbers in parentheses (saccular aneurysm and iron positivity) correspond to percentages of the total number of cerebral aneurysms in that group. P-values were calculated by Mann-Whitney U test, except for iron positivity (Fisher’s exact test). IONP-ICG was accumulated in the cerebral aneurysms with large number of macrophages. IONP-ICG, iron oxide nanoparticles (IONPs) conjugated with indocyanine green (ICG).*

## Discussion

Using an activatable IONP-ICG NIRF probe, the present study successfully detected localized macrophage-rich CA-associated chronic inflammation in rats. Specifically, histological findings showed a strong correlation between NIRF signals in the CA wall and macrophages-rich regions. Furthermore, iron positivity in the same area indicates that NIRF signals were derived from the IONP-ICG administered to live rats. In other words, intravenously administered IONP-ICG was taken up by macrophages accumulating in the CA wall, leading to activation of quenched IONP-ICG fluorescence. Thus, we concluded that macrophage localization can be determined in CAs through NIRF imaging.

The quenching of fluorescence probably occurred via the interaction between ICG and IONP (energy transfer) (Ogawa et al., 2009; Sano et al., 2013). Although the detailed mechanism on fluorescence activation was not clarified in this study and our previous report (Ikeda et al., 2018), we speculated the fluorescence could be activated when the distance between ICG molecule and IONP was increased after being processed in the lysosomes. INOPs are taken by macrophages and retained in lysosomes (Muller et al., 2007).Therefore, we speculated that IONP-ICG was activated in lysosomes and emitted fluorescence like the previously reported target cell-activatable probe (Ogawa et al., 2009; Urano et al., 2009).

As mentioned above, IONPs decrease magnetic resonance imaging signals in the aneurysm wall when taken up by macrophages (Hasan et al., 2012; Aoki et al., 2017b). A previous report showed that acetylsalicylic acid was able to inhibit IONP-induced decreases in magnetic resonance signal intensity by reducing the content of macrophages in the aneurysm wall (Hasan et al., 2013). Thus, quantitative evaluation of NIRF signals in the CA not only facilitates identification of macrophage localization sites, but also provides an index of macrophage content therein. In the present study, signals were only detected in two CAs in the IONP-ICG group, suggesting that IONP-ICG NIRF detection is associated with a greater accumulation of macrophages in the CA wall. On the other hand, the number of macrophages was significantly smaller in CAs in which signals were too low to detect by NIRF imaging.

In general, macrophages are categorized into two subsets: classically activated M1 macrophages that demonstrate pro-inflammatory effects and alternatively activated M2 macrophages that exhibit anti-inflammatory effects. Development of CAs is characterized by heightened polarization of M1 macrophages (Shao et al., 2017). Based on a previous *in vitro* experiment demonstrating that divergent differentiation of macrophages into M1 or M2 subsets does not affect uptake of IONPs (Aoki et al., 2017b), NIRF localization in the present study appears to reflect macrophage localization in general, with no predilection for different subsets. However, a mixture of macrophages with and without NIRF signal was evident within the same cryosection of NIRF-cases 1–3 (Figure 4), indicating that the IONP-ICG uptake capacity of macrophages may differ partially depending on the macrophage subset, extent of activation, or extent of accumulation.

The left posterior cerebral artery of NIRF-case 3 in the current study did not exhibit CAs or disruptions of the internal elastic lamina. However, NIRF signal was detected at the bent portion of the left posterior cerebral artery, with macrophage accumulation in the artery wall adventitia, indicating the accumulation of macrophages with a high IONP-ICG uptake capacity. In our CA rat models, the left common carotid artery was ligated which caused hemodynamic stress in the left posterior cerebral artery, representing collateral circulation for areas perfused by the left common carotid artery. The four saccular CAs at the left posterior cerebral artery that experienced hemodynamic stress were significantly larger and showed thicker walls compared to bulging CAs. This is attributable to the presence of numerous iron-positive macrophages, which could contribute to CA enlargement. Therefore, in NIRF-case 3, IONP-ICG was taken up by macrophages which could induce inflammatory responses in the left posterior cerebral artery, suggesting that early detection of a pathological stage prior to disruption of the internal elastic lamina that ultimately transforms into a CA.

Macrophages play a major role in chronic inflammation of CAs and are thought to be associated with their formation, enlargement, and rupture. Anatomical factors such as aneurysm size, shape, and site of occurrence are important for evaluating the rupture risk of unruptured CAs and determining whether surgical procedures should be performed (Morita et al., 2012). Moreover, while size is a primary risk factor for rupture, small CAs can enlarge over time, potentially resulting in rupture and subarachnoid hemorrhage. Macrophage-based imaging may enable visualization of clinically crucial aspects, namely the formation and enlargement of CAs, as well as the localization of lesions that may rupture. CAs treated with radical surgery under craniotomy occasionally lead to re-enlargement and subarachnoid hemorrhage (Tsutsumi et al., 2001; Hokari et al., 2016). By visualizing the inflammatory response in CAs (i.e., macrophage localization), intraoperative detection of artery walls that require treatment may be possible in real-time, which would improve the provision of surgical support and curability of CAs. Furthermore, macrophage-based imaging of CAs would enable three-dimensional visualization of the spread of CA inflammation. Hemodynamic stress on the endothelial cells of the cerebral artery triggers excessive inflammation in the artery wall, becoming a factor contributing to the induction of CAs (Kataoka, 2015). Inflammatory response as well as hemodynamic stress at the aneurysm wall has been suggested to be involved in the development, enlargement, and rupture of CAs, although no studies thus far have evaluated these factors in the same CA. NIRF imaging of the inflammatory response at the aneurysm wall and simultaneous quantitative evaluation of the hemodynamic stress using three-dimensional simulation through computational fluid dynamics (Sforza et al., 2009) may enable evaluation of the association between inflammatory response and hemodynamic stress. This in turn could lead to further elucidation of the pathology of CA development, enlargement, and rupture.

In the present study, NIRF signals were localized to macrophage-rich regions; however, iron staining was mostly negative at the CAs or the bent portion of the left posterior cerebral artery where ICG fluorescent signals were detected. This result was similar to that in atherosclerotic plaques shown in our previous study (Ikeda et al., 2018). It is possible that the sensitivity of iron staining might be insufficient due to relatively fewer infiltrating macrophages in these regions. Moreover, since iron staining is unable to detect IONPs prior to their degradation (Levy et al., 2011), it is possible that waiting more than 48 h after IONP-ICG injection before animal sacrifice and sample analysis would produce different results.

Several limitations to this study must be considered. First, CAs were examined *ex vivo* because it was rather difficult to expose and image CAs while *in vivo*. Because NIRF signals of CAs could be detected even with the brain in the background (Figure 3) and the NIRF signal intensity of blood was extremely low (Figure 1B), NIRF signals can likely be detected in the CA under *in vivo* conditions. However, the effects of the pulse in the CA and cerebral artery under actual *in vivo* conditions need to be taken into account. Second, since NIRF imaging in animal CA models is not entirely the same as that in humans, further examinations are needed to clarify whether the present results are applicable to human CAs. The dose of IONPs per body weight needed to visualize CA macrophages is considered to differ significantly between animals (50 mg ferumoxytol/kg) and humans (2.5–5 mg ferumoxytol/kg) (Hasan et al., 2012; Aoki et al., 2017b). Furthermore, since the conjugation of fluorescent molecules to IONP may affect the *in vivo* kinetics (Arami et al., 2015), determination of the appropriate dose and *in vivo* kinetics of IONP-ICG for visualizing CA macrophages in humans is needed. Third, the IONP-ICG group in this study was small (eight rats with 13 CAs), indicating the necessity for caution when interpreting statistical results.

In the present study, we demonstrated the potential of an activatable IONP-ICG NIRF probe for imaging of macrophages that accumulate in CAs. The individual reagents used (IONPs, ICG, PEG) are highly biocompatible and approved for clinical use by the United States Food and Drug Administration (Fruijtier-Polloth, 2005) indicating that the synthesized IONP-ICG not approved by the United States Food and Drug Administration would also have good biocompatibility. Although studies to confirm the safety of IONP-ICG are necessary before use in humans can be considered, the possibility of its clinical application in NIRF imaging for CA macrophages in humans is anticipated.

## Conclusion

Using activatable IONP-ICG NIRF imaging, we for the first time succeeded in detecting the macrophage-rich regions in CAs and/or the cerebral artery wall, which is considered an early lesion in the process of CA development.

## Data Availability Statement

The datasets analyzed in this manuscript are not publicly available. Requests to access the datasets should be directed to rocky@kuhp.kyoto-u.ac.jp.

## Ethics Statement

The animal study was reviewed and approved by Kyoto University Animal Care Committee.

## Author Contributions

HI, AI, and KS conceived and designed the study. HI and KS performed the experiments and wrote the manuscript. HI analyzed the data. All authors read, reviewed, edited and approved the manuscript.

## Conflict of Interest

The authors declare that the research was conducted in the absence of any commercial or financial relationships that could be construed as a potential conflict of interest.
